# Substructure determination using phase-retrieval techniques

**DOI:** 10.1107/S2059798317014462

**Published:** 2018-02-01

**Authors:** Pavol Skubák

**Affiliations:** a Biophysical Structural Chemistry, Leiden University, PO Box 9502, 2300 RA Leiden, The Netherlands

**Keywords:** substructure determination, single-wavelength anomalous scattering, phase retrieval, charge flipping, relaxed averaged alternating reflections, *PRASA*

## Abstract

Thus far, the application of phase-retrieval methods in crystallography has mainly been aimed at variants of charge flipping or structure-factor flipping. In this work, the relaxed averaged alternating reflections (RAAR) algorithm is applied to determine anomalously scattering substructures from single-wavelength anomalous diffraction (SAD) data of macromolecules. The algorithm has been implemented in a new program, *PRASA*, and has been shown to significantly outperform charge flipping in determining anomalously scattering substructures on a test sample of 169 SAD data sets with resolutions up to 3.88 Å.

## Abstract 2

The relaxed averaged alternating reflections (RAAR) phase-retrieval method has been applied to crystallography for the first time and has been shown to outperform charge flipping in anomalous substructure determination.

## 1. Introduction   

Rapid progress in both instrumentation and computational methods of macromolecular imaging has led to unprecedented growth in the number of macromolecular structures solved: the number of structures deposited in the Protein Data Bank (PDB; Berman *et al.*, 2000 ▸) has increased by an order of magnitude in the new millennium, with the majority of these PDB entries being solved by X-ray crystallography. Owing to the rapidly growing number of known structures, molecular replacement (MR), a technique to determine the structure under study using similar previously determined folds, has become the most frequently used technique to solve the phase problem in macromolecular X-ray crystallography: over two thirds of the X-ray crystallographic structures deposited in the PDB were solved by MR or by a combination of MR with experimental phasing techniques.

However, while MR is the apparent method of choice for many structure determinations, experimental phases remain essential in more complicated cases. Single-wavelength anomalous diffraction (SAD; Hendrickson & Teeter, 1981 ▸; Wang, 1985 ▸) is the primary method for experimental phasing, thanks to its simplicity and to advances in SAD data collection and software (as summarized by Rose & Wang, 2016 ▸). Determination of the atomic positions of the anomalously scattering substructure, composed of S, P, halogen, metal or Se atoms, from the anomalous data is the crucial first step of the method.

Most programs for SAD substructure determination, such as *SHELXD* (Schneider & Sheldrick, 2002 ▸), *SnB* (Weeks & Miller, 1999 ▸) and *HySS* (Grosse-Kunstleve & Adams, 2003 ▸), are based on the ‘direct’ methods that were originally developed for the structure solution of small molecules and that obtain phase estimates from relations between the intensities and the phases of the reflections. Direct methods are typically implemented within an iterative dual-space recycling (Weeks *et al.*, 1993 ▸) between the crystal space and reciprocal space, with prior information being used to modify the crystal space density.

### 1.1. Phase-retrieval methods   

From a more general point of view, the X-ray crystallo­graphic phase problem belongs to the class of nonlinear and nonconvex inverse problems, which have been studied intensively for decades. Although no general solution is known, in the special case of optical phase retrieval efficient algorithms have been developed which have successfully been used for reconstruction of the unknown phases in, for example, astronomical imaging (see, for example, Dainty & Fienup, 1987 ▸) and single-particle imaging (see, for example, Miao *et al.*, 1998 ▸).

Almost three decades ago, Millane summarized the similarities and differences between optical phase-retrieval approaches and the traditional crystallographic approaches to the phase problem, and suggested the application of phase-retrieval techniques in crystallographic algorithms (Millane, 1989 ▸). Despite this, the use of phase-retrieval methods for *ab initio* phasing only gained considerable interest in the crystallographic community in 2004, when Oszlányi and Sütő showed that charge flipping, one of the simplest phase-retrieval methods, can phase many high-resolution X-ray diffraction data sets (Oszlányi & Sütő, 2004 ▸); they subsequently further improved the performance of the charge-flipping algorithm (Oszlányi & Sütő, 2008 ▸).

The implementation of the charge-flipping algorithm in the program *Superflip* (Palatinus & Chapuis, 2007 ▸) showed that charge flipping can provide added value to the traditional direct methods used for X-ray crystallographic structure solution of small molecules (van der Lee, 2009 ▸). Finally, Dumas and van der Lee showed that charge flipping as implemented in *Superflip* can also be used for substructure determination from anomalous data (Dumas & van der Lee, 2008 ▸).

Similar to most current direct-methods implementations, the phase-retrieval techniques perform iterative dual-space recycling. However, unlike direct methods, which attempt to estimate the phases in reciprocal space, the operations performed by phase retrieval in either of the spaces alone cannot, even in principle, solve the phase problem (Palatinus, 2013 ▸). Constraints based either on the data or on prior information, that do not directly model or gain phase information, are applied in both spaces.

In reciprocal space, the constraints are typically given by the observed data. In crystal space, the prior information used includes non-negativity, atomicity, continuity or knowledge about the density in specific regions. The phase-retrieval algorithms differ in the way that the constraints are applied in both spaces, ranging from a simple projection of the constraint to complex transformations improving the convergence properties.

This paper reports a new adaptation of the charge-flipping algorithm for the problem of substructure determination from SAD data, which has been tested on a large set of SAD data sets. Furthermore, it reports the adaptation of the relaxed alternating averaged reflection algorithm (Luke, 2005 ▸) and its testing on the same sample of SAD data sets and shows that it outperforms the charge-flipping algorithm.

## 2. Methods   

### 2.1. Phase-retrieval algorithms for substructure determination   

Phase-retrieval algorithms can generally be described as an iterative density-modification technique in which the electron density in cycle *n* + 1 is obtained by applying an operator Θ to the current electron density ρ_*n*_:
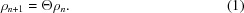
The operator Θ is composed of forward and inverse Fourier transformation operators 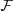 and 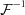, and crystal-space and reciprocal-space modification operators Θ_*Di*_ and Θ_*Mi*_, respectively. In the simplest case, a single-crystal space operator and a single reciprocal-space modification operator are applied and the index *i* can be removed:
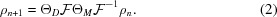



The operators Θ_*D*_ and Θ_*M*_ incorporate the information from the data and prior information in crystal and reciprocal space, respectively. In the most intuitive approach, Θ_*D*_ and Θ_*M*_ are constructed as direct projections of the constraints provided by the data and prior information. For substructure determination, the prior information of non-negativity and atomicity of the electron density can be used as a prior space information constraint in crystal space,
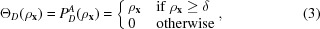
where δ ≥ 0 imposes the non-negativity and a large value of δ only retains the large electron density with an increased likelihood of corresponding to the atom peaks, thus imposing a weak atomicity constraint for the mostly flat substructure electron-density maps.

The reciprocal-space data projector can be be applied by replacing the calculated structure-factor amplitudes with the amplitudes derived from the observed data while keeping the phases unchanged,
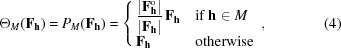
where *M* is the set of reflection indices **h** for which intensities have been measured and **F_h_** ^o^ denotes the structure-factor amplitude for the reflection with Miller indices **h** obtained by truncation of the observed intensities. In practice, the amplitudes **F_h_** ^o^ are often replaced by normalized *E* values **E_h_** ^o^.

Direct application of the projectors in a phase-retrieval iteration
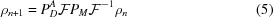
is known in crystallography as low-density elimination (LDE; Shiono & Woolfson, 1992 ▸). This algorithm has primarily been used for phase improvement by the authors; however, they also noted that it could be used for the *ab initio* solution of simple structures. Oszlányi & Sütő (2008 ▸) considered LDE to be a useful method for the optimization of *ab initio* structures of small molecules solved by charge flipping.

Generally, phase-retrieval methods directly applying data and prior constraints as projections are more suitable for the refinement of partial solutions than for solution from random phases, owing to their small radius of convergence. The radius of convergence can be improved by the incorporation of perturbation, which is typically achieved by use of a reflector operator *R* instead of the projector *P*, 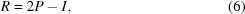
where *I* is an identity operator. The crystal-space reflector derived from the projector (4) then flips the low electron-density values around 0:
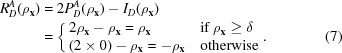
Charge flipping is a phase-retrieval algorithm using the reflector *R_D_^A^* and the projector *P_M_* (Oszlányi & Sütő, 2004 ▸):
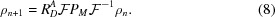



Further perturbation and thus a potentially larger radius of convergence can be achieved by application of a reflector in reciprocal space:
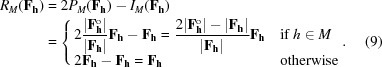
Unfortunately, simultaneous application of reflectors in both crystal space and reciprocal space suffers from instability and divergence. However, the scheme can be stabilized by ‘averaging’ with the identity operator, leading to the alternate averaging reflections (AAR) phase-retrieval method (Bauschke *et al.*, 2004 ▸; Oszlányi & Sütő, 2011 ▸):
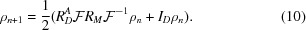
However, the AAR algorithm still tends to diverge from the solution (see, for example, Marchesini, 2007 ▸) for inconsistent problems; that is, problems for which no solution that exactly satisfies the applied constraints and data exists. Clearly, the problem of substructure determination from weak anomalous signals is strongly inconsistent owing to the tiny signal-to-noise ratio of the data. Further stabilization and improvement of the convergence properties, especially for inconsistent problems, can be achieved by the addition of a relaxation term of a crystal-space projection, with the terms weighted by a newly introduced parameter β:
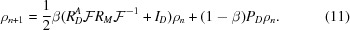
This is the iteration scheme of the relaxed averaged alternating reflections (RAAR) algorithm (Luke, 2005 ▸). The algorithm has been suggested as an interesting alternative to established schemes by Palatinus (2013 ▸), but thus far it has not been tested in a crystallographic context.

### 2.2. Implementation and testing   

The RAAR algorithm (11) was adapted to the substructure-determination problem in a new program for phase retrieval of anomalously scattering atoms: *PRASA*. The program also implements charge flipping (8), against which the RAAR algorithm is compared in this paper. Thus, the implementation is based on projector and reflector operators (3), (4), (7) and (9) as defined in the previous section. Although other algorithms and other projector operators were also tested within the new program, none of them were found to be systematically better and thus they have not been included in the implementation. The program was written in the C++ programming language and uses the CCP4 Clipper libraries (Cowtan, 2003 ▸) for general crystallographic functionality, the FFTW3 or FFTW2 libraries (Frigo & Johnson, 2005 ▸) for the fast Fourier transform operations and OpenMP for parallelization.

To determine an unknown substructure, *PRASA* starts from a map generated using the input substructure-factor amplitudes and random phases. Tests showed that rather than waiting for complete convergence of the phase-retrieval iteration scheme, a solution was usually more rapidly obtained by stopping after several hundred phase-retrieval iterations and starting another trial from new random phases. Typically, not all trials converge to the ‘correct’ solution, and the Pearson correlation coefficient (CC) between the calculated structure-factor amplitudes and the observed amplitudes is used as a quick and effective solution-selection criterion. The substructure is then obtained as the positions of peaks above 4.5σ in the density map from the trial with the largest CC.

The correlation coefficient is not only used as a relative measure to select the ‘best’ substructure from the different trials but also as an absolute measure of success: the substructure determination can be stopped if the correlation coefficient value indicates that a solution has been found. Currently, a value of 40 is used as a conservative default threshold for early termination. However, for many data sets with weaker anomalous signals a correct solution can be obtained even if the correlation coefficient is much smaller. Therefore, a quick phasing by *REFMAC*5 (Murshudov *et al.*, 2011 ▸) is performed for certain prospective solutions with CC > 10 and an early termination is also performed if CC × FOM × SCC × 100 > 40, where FOM is the reciprocal-space figure of merit after phasing and SCC is a score derived from a correlation of the experimental density map with its local r.m.s. for both hands, as calculated by the *MAPRO* utility from the *CCP*4 crystallographic package (Winn *et al.*, 2011 ▸).

Since the anomalous signal often extends to lower than the overall data resolution, a high-resolution cutoff is typically applied to the data before they are input to anomalous substructure-detection programs. Substructure determination may be very sensitive to the high-resolution cutoff parameter: especially for data sets with a weak anomalous signal, the convergence to the solution may be hindered either by the inclusion of high-resolution reflections with noise masking the anomalous signal, or by their exclusion if, in contrast, their anomalous signal prevails over the noise.

**Figure 1:**
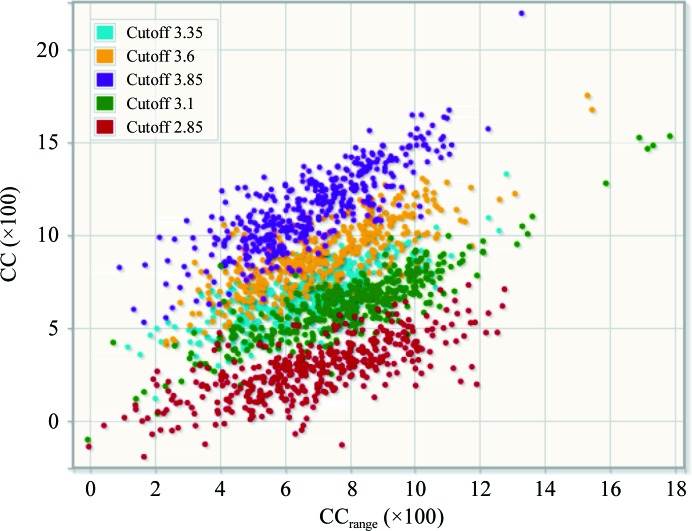
An example of *PRASA* output using multiple resolution cutoffs. A solution was obtained for three of the five tested cutoffs. Since a larger resolution cutoff generally leads to a larger CC (the different colour clusters are layered from the largest cutoff at the top to the smallest at the bottom), CC_range_ is used to score the best solutions from different cutoffs. The order in the legend corresponds to the order in which the jobs were run by *PRASA*, starting in the middle of the range.

Although the anomalous resolution can by estimated from CC_1/2_^anom^ (Karplus & Diederichs, 2012 ▸; Evans & Murshudov, 2013 ▸) or other statistics, it may still differ considerably from the optimal high-resolution cutoff for obtaining the substructure. Therefore, *PRASA* attempts to run phase-retrieval trials at several different high-resolution cutoffs: by default up to five cutoffs are used, spanning a range of up to 1 Å. The correlation coefficient is resolution-dependent and tends to increase with an increasing high-resolution cutoff, as illustrated by Fig. 1 ▸. Therefore, the ‘best’ substructure solution for each resolution cutoff *c* is first determined using the usual correlation coefficient calculated to the given resolution cutoff, denoted as CC_c_. Afterwards, the ‘best’ substructures *s*_1_, …, *s* *_N_* from the different cutoffs *c*_1_, …, *c* *_N_* are scored using CC_range_, an average of all correlation coefficients of the solution over the tested range, 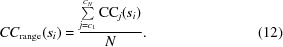



Although *PRASA* has been written as a standalone program with many command-line options, it has also been integrated in the *CRANK*2 suite (Skubák & Pannu, 2013 ▸) for macromolecular structure solution from experimental phases. In this paper, the complete *CRANK*2 solution pipeline from *F*_A_ estimation to model building was performed on 169 SAD data sets from 157 different macromolecular structures. The test sample primarily consisted of the data sets used in Skubák & Pannu (2013 ▸), which have been further extended with more recent data sets. The sample provides a wide range in terms of resolution, from 0.94 to 3.88 Å, and anomalous scatterers, such as Se, S and halogen atoms and many different heavy metals. Many of the data sets were originally solved by more complex experiments in which the SAD data were combined with other data sets (such as MAD, SIRAS and MR-SAD), and thus may be difficult to solve by SAD only. The complete list of PDB codes is provided in *Appendix A* [App appa].

The measured data provide amplitudes of structure factors corresponding to the entire macromolecule. However, to determine the substructure we need the amplitudes of structure factors corresponding to the substructure only: the *F*_A_ values. For the purpose of this work, the simplest estimation of the *F*_A_ values as the absolute value of Bijvoet differences, *F*_A_ = |*F* ^+^ − *F* ^−^| = Δ*F*, was used. The *F*_A_ values were further normalized to the *E*_A_ values using the program *ECALC* (Ian Tickle, unpublished work) from *CCP*4.

A simple *E*_A_ exclusion scheme was implemented in *CRANK*2 based on the ratios *F*_A_/*F* (with a threshold of 1) and σ(*F* ^+^)/σ(*F* ^−^) (thresholds of 1/3 and 3). All of the *E*_A_ values from *ECALC* that passed the exclusion criteria were then inputted to the *PRASA* program. Furthermore, a more advanced *F*_A_ estimation and exclusion by *SHELXC* (Sheldrick, 2015 ▸) was also tested for the data sets where *PRASA* did not succeed in finding the substructure from the *E*_A_ values prepared in the simple way described above. In the *SHELXC*–*PRASA* pipeline, the *F*_A_ factors are estimated and excluded by *SHELXC* and the corresponding *E*_A_ values converted by *ECALC* are input to *PRASA*.

The charge-flipping parameter δ was set to 1.3σ and the RAAR δ parameter was set to 3.1σ, where σ is the standard deviation of the electron-density map. However, for both algorithms the δ parameter was automatically decreased if the Fourier space iterations of the first trials diverged. The relaxation parameter β of the RAAR algorithm was fixed at 0.82. This value was chosen in initial testing on a set of training data sets that were not included in the test sample.

Furthermore, for the data sets that succeeded with RAAR but failed with charge flipping, a series of charge-flipping tests with δ varying between 1.0σ and 1.4σ with a step of 0.05σ were performed, with the automatic decrease of δ disabled. All other parameters and options were kept the same in the charge-flipping and RAAR tests. A total of 2000 trials, with 200 Fourier iterations per trial, were run for each test.

For each data set, the substructure obtained from *PRASA* is compared with the ‘final substructure’ using the program *SITCOM* (Dall’Antonia & Schneider, 2006 ▸). If available, the ‘final substructure’ was obtained from the PDB-deposited coordinates, otherwise the atomic coordinates obtained from anomalous difference maps were used. For the purposes of matching, the determined substructure is ordered by the height of the density peaks of the atoms and the end of the ordered list is cut off either at 20% of the height of the largest peak or at the number of the deposited atoms plus one, whichever leads to a smaller length of the list. The resulting fraction of correctly determined substructure is used as a measure of success of substructure determination.

Another measure of success is the ability to build the model from the *PRASA* substructures: the fraction of the protein model correctly built by *CRANK*2 is reported for all 169 data sets. The default *CRANK*2 solution pipeline was used, with *REFMAC*5 employed for the reciprocal-space processes of phasing, phase combination in density modification and phased refinement using the appropriate multivariate SAD functions. The *CCP*4 programs *Parrot* (Cowtan, 2010 ▸) and *Buccaneer* (Cowtan, 2006 ▸) are used by *CRANK*2 for real-space density modification and model building, respectively, within the ‘combined’ building algorithm (Skubák & Pannu, 2013 ▸). The input SAD data, the protein sequence and the substructure atom type and its anomalous scattering coefficients were provided as input to all of the jobs. Furthermore, the number of monomers in the asymmetric unit was input for a few data sets where the correct number significantly differs from the automatic *CRANK*2 estimation based on Matthews coefficients.

The model-building performance is judged by the fraction of the PDB-deposited model backbone that is ‘correctly built’. A residue is considered to be correctly built if its C^α^ position is at a distance of at most 2 Å from a deposited model C^α^ (‘C^α^-deposited’) position and a neighbouring C^α^ position is at a distance of at most 2 Å from a neighbour of the C^α^-deposited position.

## 3. Results and discussion   

**Figure 2:**
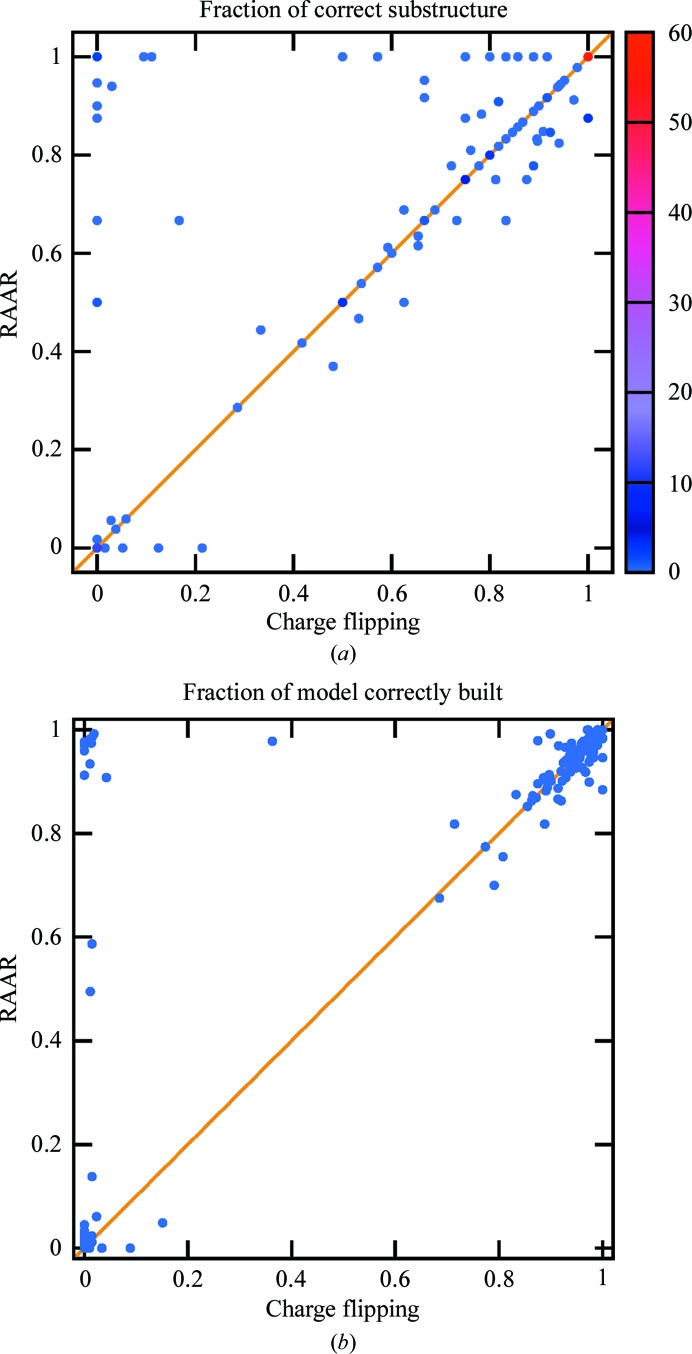
Fraction of (*a*) the substructure and (*b*) the protein backbone correctly determined by the *CRANK*2 pipeline using the RAAR and charge-flipping algorithms implemented in *PRASA*. Each light blue point in the graph represents a single data set. Since a larger number of data sets can share the same substructure-detection results, a colour gradient has been added to indicate the number of data sets behind the same dot that share the same substructure-detection results.

Fig. 2 ▸ shows the performance of *PRASA* in terms of substructures determined and macromolecular models built for the 169 SAD data sets. Owing to the ability of the ‘combined’ building algorithm to complete partial models, almost all of the resulting models can be divided into two distinct categories: either correctly built close to completion (more than 75% of the backbone correctly traced) or not built (less than 25% of the backbone correctly traced). As can be seen from Fig. 2 ▸(*b*), three models fall outside these categories: in two cases the limiting factor behind the partial (50 and 69% complete) models was the low resolution of the data set (3.88 and 3.2 Å, respectively), while the remaining data set, which was built to 59%, suffered from twinning. For the sake of simplicity, the few partially built models will be considered as correctly built in the following text.

Within this classification, the substructures determined using the charge-flipping algorithm led to 130 correctly built models and the RAAR algorithm enabled automatic building of 142 models. There were no models that could be built only by the pipeline using the charge-flipping algorithm; however, 12 models in the upper left corner of Fig. 2 ▸(*b*) could only be built by the pipeline with the RAAR algorithm.

According to the *SITCOM* analysis, no correct models could be built if less than 35% of the heavy atoms were correctly determined by *PRASA*. However, a few incomplete substructures, identified to around 40–50%, could either already be completed by *CRANK*2 or sufficed for successful phasing without completion. Thus, similarly to the binary classification of model building, substructure determination can be considered to be successful if more than 35% of the heavy atoms were found and unsuccessful if a smaller or no fraction was correctly identified. However, the class of data sets with substructures determined is not identical to the class of data sets with models built: for six data sets, the model could not be automatically built despite the substructure being identified, owing to very poor experimental maps which could not be sufficiently improved by density modification and modelling.

**Figure 3:**
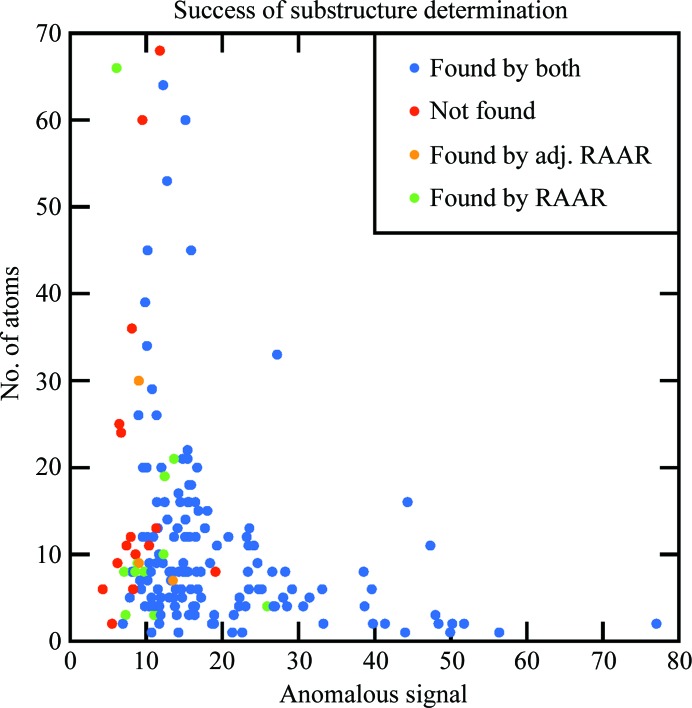
Classification of the results as a function of the anomalous signal and the number of substructure atoms. The substructures determined by both the RAAR and charge-flipping algorithms are shown in blue, unsolved substructures are shown in red, substructures determined by RAAR but not by charge flipping are shown in green, and the orange colour indicates substructures for which both algorithms failed initially but that could be solved by RAAR in an additional larger number of trials (adj. RAAR).

Similarly to the model-building evaluation, 12 more sub­structures could be determined using the RAAR algorithm compared with the charge-flipping algorithm, as shown in the upper left corner of Fig. 2 ▸(*a*). Since charge flipping is known to be strongly dependent on the δ parameter and its optimal value may vary between data sets, a series of tests with δ varying between 1.0 and 1.4σ with a step of 0.05σ was performed to find out whether charge flipping could succeed with a different δ parameter. Although δ parameters of 1.25 and 1.35σ indeed led to the heavy atoms being correctly identified in two cases, charge flipping still failed for the remaining ten data sets. Furthermore, the flip-mem variant of charge flipping (Oszlányi & Sütő, 2008 ▸) with the β parameter set to 0.6, 0.8 or 1.0 also did not lead to solution of these ten data sets. Based on these results, we can conclude that RAAR significantly outperformed charge flipping. As Fig. 3 ▸ demonstrates, the majority of the ten data sets are characterized by a lower anomalous signal. Thus, it appears that the RAAR algorithm extends the limits towards data sets with weaker anomalous signals.

The RAAR algorithm succeeded in obtaining the heavy-atom substructure for a total of 148 SAD data sets and failed for the remaining 21 data sets. However, it turned out that another three substructures could be determined by either RAAR or charge flipping if *F*_A_ values from *SHELXC* were used, proving the importance of *F*_A_ input for the determination of anomalously scattering atoms. Furthermore, a further three substructures could be determined if the number of RAAR trials was also increased from the default 400 trials per resolution cutoff to 10 000.

The heavy atoms for the remaining 15 data sets could not be found by *PRASA*. No solutions were found for these data sets in additional tests with 10 000 *SHELXD* trials per resolution cutoff, run with the same resolution cutoffs and with the other parameters set to the default for the *SHELX* pipeline implemented in *CCP*4*i*2. Although it is possible that some substructures could be still determined by further adjusting the parameters, this provides an indication that the RAAR algorithm is competitive with the ‘traditional’ state-of-the-art substructure-determination algorithms. A thorough comparison of the performance of the different approaches performed by an independent expert would be required to confirm this hypothesis.

*Ad hoc* attempts to find the substructure for the remaining 15 difficult data sets by adjustment of the β and δ parameters of the RAAR algorithm were not successful. However, a systematic search through the (β, δ) parameter space was not performed. The *ad hoc a posteriori* tests further suggested that values of β of between approximately 0.81 and 0.83 indeed appeared to be optimal if the δ parameter was set to values around 3σ. However, good results could be also obtained for other combinations of these two parameters.

As Fig. 3 ▸ shows, the success of substructure determination unsurprisingly depends on the strength of the anomalous signal. Here, the anomalous signal is estimated from the average peak height in the anomalous difference maps, phased using the ‘best’ phases corresponding to the deposited PDB models, at the positions of anomalous substructure atoms (see, for example, Yang *et al.*, 2003 ▸; Terwilliger *et al.*, 2016 ▸). Using the RAAR algorithm, all of the substructures were found with a peak height larger than 12σ, except for the 2prx data set, which turned out to be surprisingly resilient to substructure-determination attempts despite a large peak height of 19σ, possibly owing to twinning of the crystal. Furthermore, the majority of substructures could still be found for anomalous signals between 8 and 12σ, with the chance of success decreasing rapidly at around 8σ. A similar conclusion was drawn by Terwilliger *et al.* (2016 ▸) for substructure detection using likelihood-based methods. It should be noted that these findings only apply to detection of the entire substructure: typically, if larger peaks of the substructure are found its smaller peaks can also be correctly located, down to around 4–5σ.

Furthermore, the testing showed that the number of substructure atoms parameter is much less important for RAAR than for current direct-space methods, where a precise estimate is sometimes crucial in difficult substructure determinations. In fact, all of the reported RAAR tests were performed without inputting the expected number of heavy atoms to be found. The reason for this behaviour is that this parameter is not directly used by the recycling algorithm. If input, it can be still used to select only the specified number of largest substructure peaks for scoring and substructure output.

An early termination of the RAAR substructure determination, before the maximal number of 2000 trials had been run, was used for 89 data sets: in 59 cases an early stop was triggered by reaching the CC × FOM × SCC threshold and in the remaining 30 cases by reaching the CC threshold. In all of these cases the solution was indeed correct and the protein model was built. A large group of the remaining data sets also provided large values of these estimators, albeit in the range that was occasionally also provided by an incorrect or incomplete substructure.

## 4. Conclusions   

In the tests on 169 SAD data sets, it has been shown that the RAAR algorithm, implemented in the new program *PRASA* for substructure determination, outperforms the charge-flipping algorithm as implemented in the same program. An analysis of the anomalous signals of the data sets solved only by RAAR indicates that the RAAR algorithm extends the limits of charge flipping towards data sets with weaker anomalous signals.

The strength of the anomalous signal remains the major limiting factor of the method, with the probability of success significantly decreasing at around 8σ. No such limitation has been found for the number of searched substructure atoms within the scope of the test sample with at most 70 substructure atoms.

Substructure determination by *PRASA* has been integrated into the *CRANK*2 pipeline for automated structure solution from experimental phases and provides features such as the automatic evaluation of multiple resolution cutoffs, early termination on success and no requirement for an estimate of the number of substructure atoms.

In the future, new phase-retrieval algorithms will be explored to further increase the radius of convergence of the method and to tackle data sets that have eluded current substructure determination. Furthermore, the possibility of the application of phase retrieval by *PRASA* to other crystallo­graphic problems, such as the phase optimization of weakly phased maps, will be investigated.

## Appendix A Complete list of PDB codes

A total of 169 SAD data sets for the following 157 macromolecular structures were used: PDB entries 1c8u, 1djl, 1dpx, 1dtx, 1dw9, 1e3m, 1e42, 1e6i, 1fj2, 1fse, 1ga1, 1hf8, 1h29, 1i4u, 1lvy, 1lz8, 1m32, 1mso, 1ocy, 1of3, 1rgg, 1rju, 1vjn, 1vjr, 1vjz, 1vk4, 1vkm, 1vlm, 1vqr, 1z82, 1zy9, 1zyb, 2a3n, 2a6b, 2ahy, 2aml, 2avn, 2b78, 2b79, 2b8m, 2etd, 2etj, 2ets, 2etv, 2evr, 2f4p, 2fdn, 2fea, 2ffj, 2fg0, 2fg9, 2fna, 2fqp, 2fur, 2fzt, 2g42, 2g4h, 2g4j, 2g4k, 2g4l, 2g4m, 2g4n, 2g4o, 2g4p, 2g4q, 2g4r, 2g4s, 2g4t, 2g4u, 2g4v, 2g4w, 2g4x, 2g4y, 2g4z, 2g51, 2g52, 2g55, 2gc9, 2hba, 2ill, 2nlv, 2nuj, 2nwv, 2o08, 2o0h, 2o1q, 2o2x, 2o2z, 2o3l, 2o62, 2o7t, 2o8q, 2obp, 2oc5, 2od5, 2od6, 2oh3, 2okc, 2okf, 2ooj, 2opk, 2osd, 2otm, 2ozg, 2ozj, 2p10, 2p4o, 2p7h, 2p7i, 2p97, 2pg3, 2pg4, 2pgc, 2pim, 2pn1, 2ppv, 2pr7, 2prr, 2prv, 2prx, 2pv4, 2pw4, 2q2l, 2rkk, 2v0o, 3bpj, 3fki, 3gyv, 3k9g, 3km3, 3lmt, 3lmu, 3men, 3njb, 3o2e, 3oib, 3p96, 3s6l, 4us7, 4xvz, 4xxt, 4yf1, 5b82, 5gwd, 5ifg, 5irr, 5j4r, 5kjh, 5lg6, 5llw, 5loi, 5lsq, 5sus, 5suu, undeposited glucose isomerase and Ca-subtilisin data sets from Dauter *et al.* (2002 ▸) and a novel undeposited data set.
